# Agglomerated loricae of the tintinnids *Codonella, Codonellopsis* and *Dictyocysta* from North Atlantic, tropical Pacific and Southern Ocean waters

**DOI:** 10.1093/plankt/fbaf030

**Published:** 2025-08-12

**Authors:** Gustaaf M Hallegraeff, Ruth S Eriksen, Karine Leblanc

**Affiliations:** Institute for Marine and Antarctic Studies, University of Tasmania, 20 Castray Esplanade, Battery Point, Hobart, TAS 7004, Australia; Institute for Marine and Antarctic Studies, University of Tasmania, 20 Castray Esplanade, Battery Point, Hobart, TAS 7004, Australia; CSIRO Environment, 3-4 Castray Esplanade, Battery Point, TAS 7004, Australia; Australian National Algae Culture Collection, CSIRO National Collections and Marine Infrastructure, 3-4 Castray Esplanade, Battery Point, TAS 7004, Australia; Aix Marseille Université, Université de Toulon, CNRS, IRD, MIO UM 110, 163 avenue de Luminy, 13288 Marseille, France

**Keywords:** tintinnids, coccoliths, *Codonellopsis* cf. *soyai*, *Codonellopsis pusilla*, *Dictyocysta lepida*, *Codonella galea*

## Abstract

Some 30 species of planktonic tintinnids agglomerate coccoliths and diatom fragments on to their loricae, which have fascinated scientists for centuries. We here provide semi-quantitative scanning electron microscopic observations on tintinnid lorica agglomerations by the small Southern Ocean *Codonellopsis* cf. *soyai* Hada, intermediate sized North Atlantic *Codonellopsis pusilla* (Cleve) Kofoid and Campbell, and larger Southern Ocean *Dictyocysta lepida* Ehrenberg and tropical Pacific *Codonella galea* Haeckel. Smaller tintinnids appeared to non-selectively use ubiquitous *Emiliania huxleyi* liths, but when absent could shift to ambient diatom fragments, while larger tintinnids preferentially used heavier coccoliths of *Calcidiscus, Coccolithus* and *Helicosphaera* adding up to an estimated 25% extra lorica weight. Lorica agglomerations appeared more prominent in colder waters compared to warmer tropical waters. Selective feeding in tintinnids is closely linked to their swimming behavior, and possible benefits from agglomerated loricae for ballasting and slowing of swimming speed are discussed.

## INTRODUCTION

Tintinnids are ubiquitous planktonic ciliates that live inside a protective solid shell, called a lorica, with diagnostic morphology. These microzooplankton create a feeding current that draws food particles toward their mouth using a crown of continuously beating oral cilia (Wandel and Holzman, 2022). Their proteinaceous loricae have fascinated scientists for centuries (Haeckel, 1873). Cementing of mineral particles for test formation is well documented also for foraminifera, phaeodarians and thecate amoebae (Stout and Walker, 1976; Gowing, 1989; Bowser and Bernhard, 1993), but tintinnids stand out for their capacity to also use coccoliths and diatom fragments. Tintinnid loricae can be either hyaline, or agglutinated with biogenic (coccoliths, diatom frustules) or non-biogenic particles (sand grains, mineral flakes). If the agglutinated particles are comprised exclusively of mineral grains the lorica is called arenaceous (“sandy”), but if decorated with particles of mineral and/or biological origin the lorica is called agglomerated (Gold and Morales, 1976). Adhering particles can cover the entire surface or only the bowl, but mostly leave the collar uncovered. Whether agglomerated particles are rejected food particles or directly captured on to sticky loricae is not clear. Details of the process of lorica formation and agglutination also remain incompletely understood. Agglutination appears to stop after construction of the bowl when the lorica hardens and loses its stickiness (Agatha *et al.,* 2013). That is, biogenic particles are captured during only a very brief time slot immediately after tintinnid cell division. In the classical taxonomic monographs on tintinnids by Kofoid and Campbell (1929, 1939) these authors illustrated by line drawings 753 tintinnid species from the eastern tropical Pacific, of which 22 species (3%) carried coccoliths and only a single species (*Laackmanniella naviculaefera*) carried diatoms. The increasing use of scanning electron microscopy (SEM) has added many more agglomerated tintinnid species to this list, but more importantly this allowed for resolution of the precise identity of agglomerating biogenic materials (Burns, 1983; Takahashi and Ling, 1984; Winter *et al.,* 1986; Wasik *et al.,* 1996; Henjes and Assmy, 2008).

Intriguingly, only selected tintinnid genera such as *Acanthostomella, Codonella, Codonellopsis, Dictyocysta, Laackmaniella*, *Stenosemella* and *Tintinnopsis* appear to be able to agglutinate biogenic particles. Furthermore, not all species within a genus agglutinate (Kofoid and Campbell, 1929, 1939). Much discussion has centered on whether the adhered particles, either food remnants or suspended particles from the water column, reflect the nature of the ecosystem in which the lorica was built. At present, only three semi-quantitative studies exist on tintinnid lorica agglomerations, two of which focused on the small tintinnid *Stenosemella* using coccoliths and/or diatoms (Winter *et al.,* 1986; Henjes and Assmy, 2008), and one study that focused on diatom agglomerations (Wasik *et al.,* 1996). In the present work, we add new semi-quantitative SEM observations from the North Atlantic, tropical Pacific and Southern Ocean Oceans: (i) to expand the list of tintinnid taxa with agglomerated loricae; (ii) identify the nature of coccolith and diatom biogenic particles on tintinnids in different ocean regions; as a first step (iii) to better understand the possible ecological advantage of lorica agglomeration.

## MATERIAL AND METHODS

### Sample collection and preparation

**Fig. 1:**
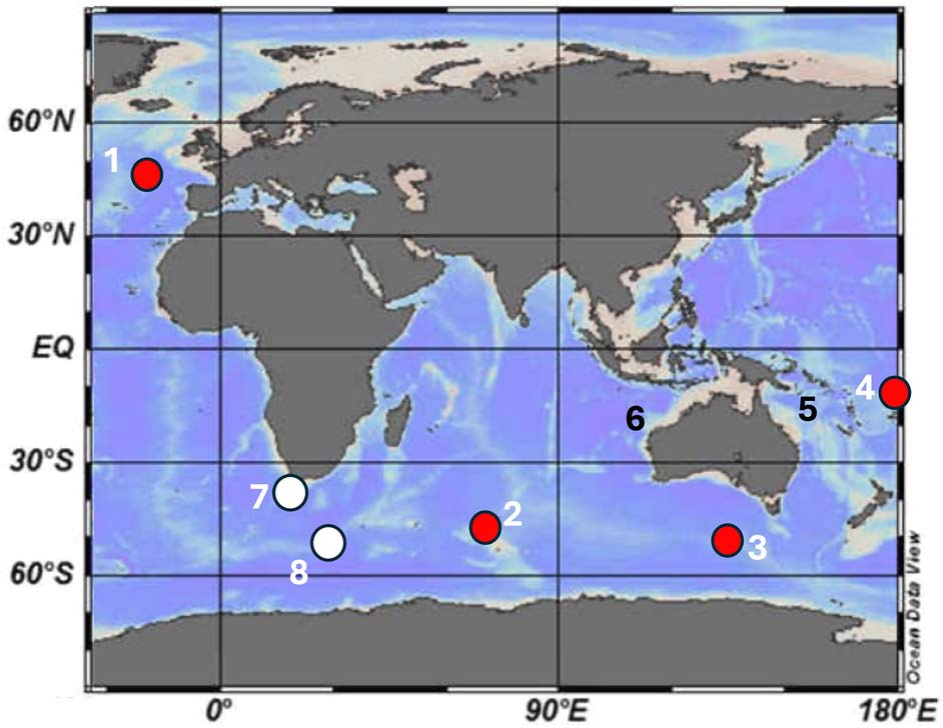
Sample locations for SEM observations of agglomerated tintinnid loricae used in the present study: 1. North Atlantic (Porcupine Abyssal Plain); 2. Kerguelen Island Plateau; 3. SOTS station; 4. Tonga-Kermadec Arc. Supplementary material was obtained from 5. The Coral Sea; and 6. Indian Ocean. The locations of previous semi-quantitative tintinnid lorica studies by 7. Winter *et al.* (1986) and 8. Henjes and Assmy (2008) are also indicated. Red dots (locations 1–4) represent the main sample materials used, black numbers (locations 5–6) supplementary material, and white dots (7–8) location of literature data.

Water samples from the Southern Ocean Time Series (SOTS) voyage were collected on board *RV Investigator* in May 2023 using a CTD rosette sampler at depths between 10 and 80 m, filtered directly on board ship on 0.8 μm Nuclepore filters, rinsed with deionized water, airdried, coated with platinum (5 nm layer thickness), and examined with a Hitachi SU70 field emission scanning electron microscope (FESEM). Samples collected using the *RV Sprightly* cruises SP16/80 and SP 3A/82 in the Coral Sea in November 1980 and March 1982, respectively, employed a 37 μm freefall plankton net from surface to 200 m depth. Unpreserved samples were collected on to polycarbonate filters on board ship, airdried and examined with a JEOL-35C Scanning Electron Microscope. The *MobyDick* cruise took place in February–March 2018 around Kerguelen Island, in the Indian sector of the Southern Ocean. Phytoplankton net and bottle net samples (Agusti *et al.,* 2015) were collected at four locations during the late summer bloom. Light microscopy images were captured on board on live samples less than 30 min after sampling, while SEM pictures were acquired 3 months later on uncoated polycarbonate filters with a Phenom SEM. Samples from the APERO campaign off the Porcupine Abyssal Plain in the North Atlantic were collected in June–July 2023 using Niskin bottles, phytonets (35 μm mesh) (from 0 to 200 m), particle traps (from 50 to 1000 m) and deep nets (20 μm mesh) from 200 to 4000 m. For each sample, two aliquots were fixed with acid Lugol and basic formalin for observation by light microscopy (Nikon TE-200) while a third sample was filtered directly on board ship on a polycarbonate filter, then coated with gold (8 nm thickness) for observation by Phenom ProX SEM. Samples on the *Tonga* cruise were collected in October–December 2019 from Niskin, phytonets (35 μm mesh) and deep nets (20 μm) (from 200 to 2000 m), and observed on a Nikon TE-200 light microscope and a Phenom Pro SEM. The scientific cruise details of the sample collections used in this study have been summarized in Table I and the geographic locations are mapped in Fig. 1.

**Table I:** *Sample locations for SEM observations of agglomerated tintinnid loricae used in the present study. Locations are mapped in Fig. 1*.

Locality	Coordinates	Dates	Sampling device	Sample preparation	Tintinnid taxa
**Tropics**					
Indian Ocean (Fig. 1, site 6)	along 110°E, 11.5–23^o^S	IIOE-2 *RV Investigator* IN2019 voyage V03 May–June 2019	20 μm net, 0–20 m depth	Lugol and buffered formalin preserved, Hitachi FESEM	*Dictyocysta lepida*
Coral Sea (Fig. 1, site 5)	SP 16/80; SP3A/82; 149–152°E, 15–21 ^o^S	*RV Sprightly* cruises SP16/80; SP3A/80 outer edge Great Barrier Reef; November 1980; March 1982	37 μm freefall net, 0–200 m depth	Unpreserved, Nuclepore filters, Hitachi FESEM	*Codonella galea, Dictyocysta lepida, Dictyocysta spinosa*
Tropical Pacific (Fig. 1, site 4)	Tonga-Kermadec Arc; 165°E–165^o^W; 19–22^o^ S	TONGA expedition October–December 2019	Niskin bottles, phytonets (35 μm mesh), deep nets (20 μm) (from 200 to 2000 m	Lugol preserved. Phenom ProX SEM	*Codonella galea*
**Temperate waters**					
North Atlantic (Fig. 1, site 1)	Porcupine Abyssal Plain; 48^o^50’N, 016^o^30’W	APERO expedition, June–July 2023	Niskin bottles, phytonets (35 μm mesh), 0–200 m, particle traps, 50–1000 m, deep nets (20 μm mesh), 200–4000 m	Unpreserved, polycarbonate filter, Phenom ProX SEM on untreated and uncoated filters	*Dictyocysta lepida, Codonellopsis pusilla*
**Southern Ocean**					
(Fig. 1, site 2)	Kerguelen Island plateau; 67–75°E, 49–52^o^S	MOBYDICK cruise February–March 2018	35 μm mesh phytoplankton net and bottle net (holding 20 μm mesh) samples	Unpreserved, polycarbonate filter Phenom ProX SEM on untreated and uncoated filters	*Codonellopsis cf. soyai*
(Fig. 1, site 3)	Southern Ocean Time Series (42.84–47.48^o^S; 139.8–147.6°E)	*RV Investigator* IN2023-voyage V03, May 2023	CTD rosette sampler,10–80 m depth	Unpreserved, Nuclepore filters, Hitachi FESEM	*Codonellopsis pusilla Dictyocysta lepida*

### Tintinnid lorica assessment

Scanning micrographs of loricae (10 to 35 per species) were printed full-size on A4 paper and individual coccoliths or diatoms contoured, counted and measured. While scanning micrographs have the advantage of resolution of the identity of biogenic materials, when loricae are too densely covered with agglutinated particles this can prevent identification of the tintinnid species. We therefore only selected loricae for which tintinnid identity was unambiguous. Similarly, SEMs typically only allow for inspection of half of the lorica surface, and we had to assume (but see Fig. 26) that both lateral sides were identical.

In order to estimate the extra lorica ballast from carrying coccoliths, we used the following conversion factors. The ellipsoidal liths of *Emiliania huxleyi* (3–4 μm length) and *Gephyrocapsa muelleri* (3.5–5 μm length) were nearly identical in size, *Syracosphaera* ellipsoidal liths ranged up to 5.6 μm long, *Coccolithus* liths were up to 7.8 μm long, the discoidal liths of *Calcidiscus* were 9 μm in diameter, and *Helicosphaera* liths were 6–12 μm long. We used coccolith weights estimated by Beaufort (2005) and Young and Ziveri (2000) to calculate the lith weights as follows: *Emiliania* 4 pg CaCO_3_ per lith, *Gladiolithus* (4 pg), *Syracosphaera* (10 pg), *Umbilicosphaera* (16 pg), *Florisphaera* (20 pg), *Rhabdosphaera* (60 pg), *Helicosphaera* (140 pg), *Calcidiscus* (124 pg) and *Coccolithus* (152 pg). Different morphotypes of *E. huxleyi* were not discriminated in these calculations. To estimate the weight of diatom fragments, we estimated their biovolumes (e.g. *Pseudo-nitzschia* fragment 25 μm^3^, *Chaetoceros* setae 40 μm^3^, *Fragilariopsis* fragment 63 μm^3^, *Thalassiothrix* fragment 100 μm^3^, *Eucampia* cribrum fragment 250 μm^3^, *Coscinodiscus* cribrum fragment 375 μm^3^), and converted biovolumes to silica using the equations of Conley *et al.* (1989); roughly 0.0005 pmole silica per μm^3^, with 1 pmole equaling to 28 pg silica. For small intact diatoms such as *Fragilariopsis pseudonana* we used a biovolume of 100 μm^3^ corresponding to 1.4 pg silica per cell.

### Water column coccolithophorids

For the APERO North Atlantic samples, semi-quantitative coccolith counts were conducted on ~0.25 mL subsamples from 1 L diluted plankton net collections, counting ¼ of a 25 mm filter by Phenom SEM. Southern Ocean (SOTS) ambient coccolithophorid counts derive from deep (1000–3800 m) sediment trap samples preserved in mercury chloride, of which subsamples were mounted on microscope slides, and coccospheres and coccoliths counted using a Nikon Eclipse 80i polarized light microscope at 1000× magnification (Rigual Hernández *et al.,* 2020.) Unfortunately, no ambient coccolithophorid data were available for the Indian Ocean, Tonga and MobyDick (Kerguelen Plateau) cruises.

## RESULTS

### *Codonellopsis* cf. *soyai*

**Figs 2–10:**
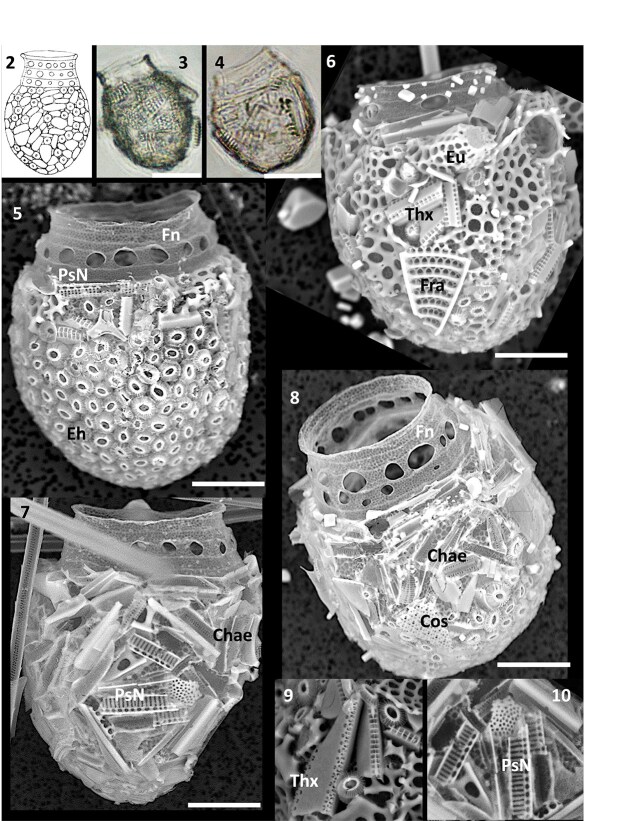
Fig. 2: Type illustration of *Codonellopsis soyai* from the Indian Ocean sector of the Southern Ocean (reproduced from Hada, 1970, Fig. 48); Figs 3–10. Tintinnid loricae of *C.* cf. *soyai* from the Kerguelen Island Plateau. Figs 3–4. Light micrographs of long and short loricae with agglutinated *Fragilariopsis* diatom fragments and whole cells; Figs 5–10. Scanning Electron Micrographs of loricae ornamented by (Fig. 5) predominantly *Emiliania huxleyi* (Eh) coccoliths or (Figs 6–8) broken diatom fragments of Cos = *Coscinodiscus/Asteromphalus*, Eu = *Eucampia*, Fra = *Fragilariopsis kerguelensis,* Thx = *Thalassiothrix antarctica*, PsN=*Pseudo-nitzschia*, Chae = *Chaetoceros* setae. Figs 9 and 10. High magnification of diatom fragment agglutination. Scale bars: 25 μm (Figs 3–4); 10 μm (Figs 6–8).

A small tintinnid closely resembling *Codonellopsis soyai* Hada described from the Indian sector of the Southern Ocean (Hada, 1970, Fig. 48; type illustration reproduced as present Fig. 2) was common off the Kerguelen Island Plateau in February–March 2018 (Fig. 1, location 2). Diagnostic features were the flask-shaped lorica composed of an ovoid bowl [33–44 μm (rarely 49–52 μm) long, 32–40 μm wide] and transparent (Fig. 3) subcylindrical collar (7.5–8.5 μm high), composed of 3–5 rings, with numerous (7–12) ovate fenestrae present mostly in the middle two rings (Figs 5–8). The surface of the obovoid bowl, 21–24 μm oral diameter, as observed on broken or corroded loricae, was smooth, but always densely covered with coccolithophorids and/or diatoms or diatom fragments. Both longer (Fig. 3) and shorter loricae (Fig. 4) co-occurred, with comparable oral diameter, but these all exhibited identical agglutination patterns.

Of 11 loricae examined, six were predominantly covered by *E. huxleyi* (Fig. 5; based on genetic arguments also referred to as *Gephyrocapsa huxleyi,*  Bendif *et al.,* 2023; Wheeler *et al.,* 2023) but five others were almost exclusively covered with diatom fragments (Figs 6–10), including broken *Chaetoceros* setae*,* fragments of *Fragilariopsis, Pseudo-nitzschia, Thalassiothrix,* and fragments of *Coscinodiscus/Asteromphalus* and heavily silicified frustules of *Eucampia antarctica.* The diatom fragments were agglutinated directly onto the loricae and difficult to quantify. Rarely, complete cells of the diatom *Fragilariopsis* were found attached (Fig. 3). For loricae dominated by *Emiliania*, the coccolithophorid load was estimated to be up to 1488 pg per lorica (Table II). For loricae covered exclusively by diatom fragments this was estimated to be much lower as 35–296 pg per lorica.

**Table II:** *Biogenic particles on loricae of* Codonellopsis soyai *from the Kerguelen Island Plateau*. Emiliania *lith cover (pg CaCO_3_/lorica) and diatom fragments (as pg silica/lorica)*.

*Emiliania* pg CaCO_3_/lorica (% of total ballast)	Diatom fragments pg silica/lorica						Total ballast pg/lorica
Kerguelen Island Plateau	*Fragila* *riopsis*	*Pseudo-nitzschia*	*Chaeto* *ceros* setae	*Thalassio* *thrix*	*Coscino* *discus* cribra	*Eucam pia* cribra	
1488 (100)							1488
880 (99)	5	0.7		5.6			891
960 (92)					84		1044
464 (98)	2	1		8			475
528(96)			13		11		552
288(97)	5	1	2				296
116(64)	2			25	32	7	180
88(56)				11	52	7	158
88(79)			13		10		111
0 (0)			15		10		25
0 (0)	26	2	7				35
445 ± 461 (73 ± 36%) (*n* = 11)	5 of 11	4 of 11	5 of 11	4 of 11	6 of 11	2 of 11	477 ± 454

### Codonellopsis pusilla

**Figs 11–18:**
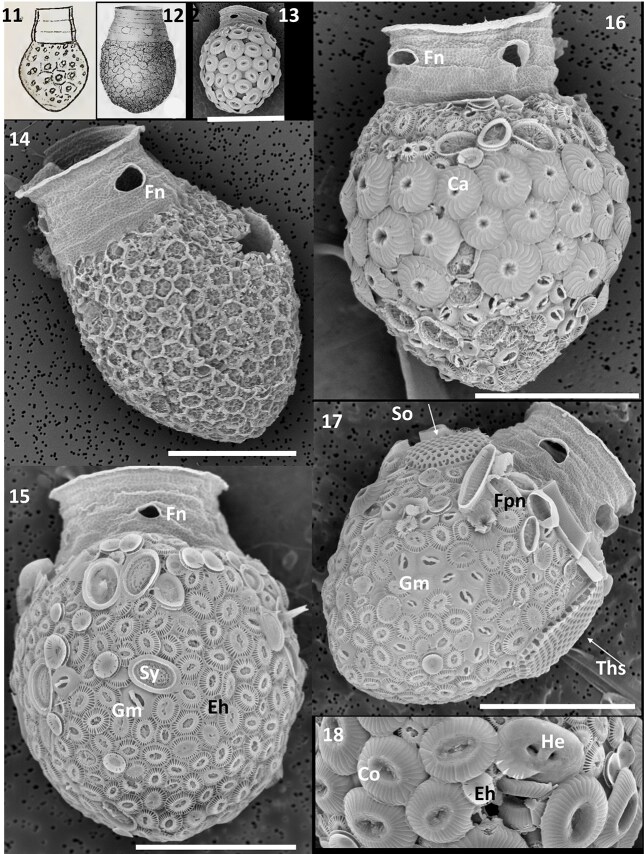
Fig. 11: Type illustration of *Codonellopsis pusilla* from Newfoundland Banks (Cleve, 1899, p.970, Fig. 3). Fig. 12: Type illustration of *Codonellopsis contracta* from the Peruvian Current showing sparse fenestrae (Fn) (Kofoid and Campbell, 1939, Fig. 147); Figs 13–18: Tintinnid loricae of *C. pusilla* from the North Atlantic (Porcupine Abyssal Plain); Scanning electron micrographs showing variations in bowl shape according to heavy coccolith agglutination (Figs 13 or 14). Empty corroded clavate bowl with hexagonal surface structure from an unpreserved sample filtered directly on board ship. Lorice ornamented by (Fig. 15) predominantly *Emiliania huxleyi* with *Syracosphaera pulchra* (Sy) and *Gephyrocapsa muelleri* (Gm); (Fig. 16) *Calcidiscus leptoporus* (Ca); and (Fig. 17) agglutinated diatoms *Shinodiscus oestrupii* (So), *Thalassiosira* (Ths) and complete diatom cells of *Fragilariopsis pseudonana* (Fpn). Fig. 18 High magnification of large *Coccolithus* (Co) and *Helicosphaera carteri* liths, compared to small *E. huxleyi* liths (Eh). Scale bars: 20 μm (Figs14–17); 30 μm (Fig. 13).

**Figs 19–24:**
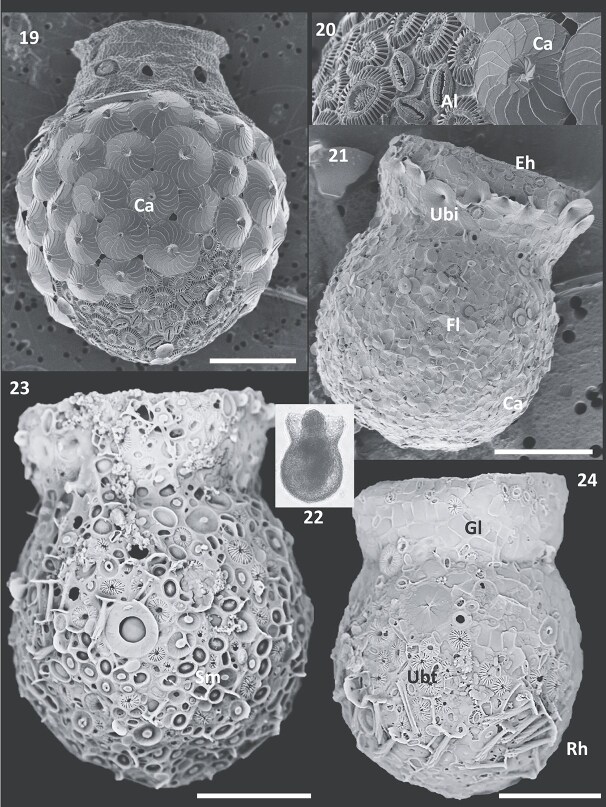
Figs 19–20: Scanning Electron Micrographs of *Codonellopsis pusilla* from the Southern Ocean, agglomerated with predominantly *Calcidiscus leptoporus* (Ca) liths, and (Fig. 20) *E. huxleyi* and *Alisphaera unicornis* (Al) liths only at the bottom of the bowl. Figs 21–24: *Codonella galea* from the Coral Sea (Fig. 21) and Tonga waters. (Figs 22–24). Fig. 21. Scanning micrograph of lorica from Coral Sea agglomerated mainly with *Florisphaera* (Fl) and sparse *Emiliania* (eh) and *Umbellosphaera irregularis* (Ubi) liths only around the collar. Fig. 22. Light micrograph of *C. galea* showing agglutinations also on the non-transparent collar. Figs 23–24. Scanning Electron Micrographs of *C. galea* from Tonga, adorned with *Umbellosphaera tenuis* (Ubt), *Syracosphaera mediterranea* (Sm), with (Fig. 24) *Gladiolithus* (Gl) around the collar large *Rhabdosphaera* (Rh) liths concentrated mostly toward the bottom of the bowl. Scale bars: 10 μm (Fig. 19); 20 μm (Figs 23 and 24); 25 μm (Fig. 21).

Tintinnids corresponding to *Codonellopsis pusilla* (Cleve) Kofoid and Campbell (type illustration by Cleve, 1899, p.970 as *Codonella pusilla* reproduced as present Fig. 11) were abundant in samples from the North Atlantic Porcupine Abyssal Plain (Fig. 1, location 1). This species had a clavate to ovate lorica (Figs 11–17), 29–35 μm wide, 32–35 μm bowl length, with a transparent collar of 4–5 rings, oral diameter 18–23 μm, and with 3–5 large elliptical fenestrae (Figs 14–17) in a single middle ring of the collar. The bowl was widest near its middle, with a hemispherical or sometimes pointed aboral end. Non-agglomerated corroded loricae exhibited a distinct hexagonal surface pattern on the lorica wall (Fig. 14) also described for the related *Codonellopsis contracta* Kofoid and Campbell (type illustration reproduced in Fig. 12). Non-agglutinated loricae were clavate in shape but heavily agglomerated loricae more ovoid or even bulbous in outline. Of 35 loricae examined from the North Atlantic, the majority were covered by different morphotypes of *E. huxleyi* coccoliths (Fig. 15)*,* with small amounts of *Syracosphaera pulchra* liths regularly present. Only five loricae were dominated by *Calcidiscus* (positioned in a distinct band; Fig. 16), three were dominated by *Coccolithus braarudii/pelagicus* (Fig. 13) with the diatoms *F. pseudonana* (4–5 μm long) on top of the bowl, with a single cell of *Shionodiscus oestrupii* or *Nitzschia bicapitata* (14 μm long; not shown) also observed embedded on top (Fig. 17). A typical lorica carried an estimated average coccolith weight of 1689 ± 542 pg, but the capture by 30% of the North Atlantic tintinnid population of *Calcidiscus, Helicosphaera* and *Coccolithus* liths (Fig. 18) raised this value by 3 to 6 fold up to 9696 pg (mean 2585 ± sd 2390 pg). The addition of small diatoms on the top of the bowl at most added only some 3% to lorica weight (Table III). Corroded loricae showed that larger *Coccolithus* and *Calcidiscus* liths did not create a canopy over smaller liths. By contrast, small *Fragilariopsis* diatoms were superimposed upon *Emiliania* liths. North Atlantic lorica agglomerated with *Calcidiscus* and *Coccolithus* liths were estimated to be heavier than those that carried accessory diatoms (Table III). Comparable loricae were also observed at the SOTS station (Fig. 1, location 3; Fig. 19, *n* = 13), oral diameter 18–19 μm, length of bowl 35–36 μm, but these were either completely covered by *Calcidiscus,* or with smaller *Emiliania* and sometimes *Alisphaera* mainly covering the 20–30% of the bottom of the bowl (Fig. 20). A single whole diatom cell of *N. bicapitata* was observed on only 1 out of 13 loricae studied. The Southern Ocean loricae, heavily ballasted by *Calcidiscus,* were estimated to weigh an extra 10 540 ± 3537 pg (Table III).

**Table III:** *Biogenic particles agglomerated on the loricae of* Codonellopsis pusilla *from the North Atlantic and Southern Ocean. Estimated as pg CaCO_3_/lorica for individual coccoliths and as total pg liths*.

	*Emiliania*	*Gephyrocapsa oceanica*	*Calcidiscus*	*Coronosphaera*	*Umbellosphaera*	*Helicosphaera*	*Coccolithus*	Total pg CaCO_3_/lorica	Diatom Fragments
*Calcidiscus* dominated-type (*n* = 10; Fig. 16)									
North Atlantic	104 ± 33	31 ± 22	35 ± 11	14 ± 7	0	0	0	5008 ± 1305	*Fragilariopsis pseudonana* 4 of 11 loricae
*Coccolithus* dominated - type (*n* = 5; Fig. 13)									
	81 ± 38	1 ± 1	0	0	0	3 ± 4	40 ± 12	6966 ± 1990	*Nitzschia bicapitata* 2 of 5 loricae
*F. pseudonana* diatom-type (*n* = 9; Fig. 17)									
	187 ± 344	19 ± 312	1 ± 31	8 ± 310	0	0	0	1228 ± 3518	34 ± 35 *F. pseudonana* pg silica per lorica
Others (*n* = 11; Fig. 15)									
	213 ± 53	146 ± 13	13 ± 13	16 ± 10	0	0	0	1276 ± 323	
Total (*n* = 35)	170 ± 70	17 ± 16	7 ± 14	11 ± 10	1 of 35	1 of 35	3 of 35	2585 ± 2390	11 of 35 loricae
Southern Ocean Time Series (*n* = 13)	50 ± 40	1 of 13	83 ± 30	1 of 13	0	0	0	10 540±3537	*N. bicapitata* 1 of 13 loricae

### Codonella galea

Loricae identified as *Codonella galea* Haeckel had a large pot-shaped body, oral diameter 40–72 μm (average ± sd of 51 ± 10 μm), 60–113 μm (77 ± 16 μm) long, divided into a funnel-shaped collar with finely denticulate margin and a subspherical bowl, separated by a constricted neck (Figs 21–24). *Codonella* differs from *Codonellopsis* in that the collar is not hyaline (Fig. 22) but mineral coated and includes agglutinated biogenic particles. It differs from *Codonaria* in the absence of a suboral cone. A single lorica from the Coral Sea (Fig. 1, location 5) was densely agglomerated with small liths resembling *Florisphaera profunda* (Fig. 21; compare Young *et al.,* 2003, plate 39, Fig. 3) with *Umbellosphaera irregularis* and *Emiliania* liths only present around the top of the collar. Liths resembling *Gladiolithus flabellatus* were found around the collar of Tonga loricae (Fig. 24; compare Young *et al.,* 2003, plate 39, Fig. 11). A distinctive feature of this tintinnid was the use of prolate *Rhabdosphaera* rhabdoliths (up to 12 μm long) on 6 out of 12 loricae from tropical Tonga (Fig. 1, location 4), mostly on the bottom of the bowl (Fig. 24), accompanied by *Umbellosphaera*, *Syracosphaera mediterranea* and sparse *Emiliania.* Coccolith loadings for this large tintinnid were low and variable in the range 1312 ± 706 pg (Table IV).

**Table IV:** *Biogenic particles agglomerated on the loricae of* Codonella galea. *Estimated as pg CaCO_3_/lorica for individual coccoliths and as total liths*.

	*Emiliania huxleyi*	*Calcidiscus*	*Calciosolenia*	*Florisphaera*	*Gladiolithus*	*Umbilicosphaera*	*Umbellosphaera*	*Syracosphaera pulchra/mediterranea*	*Rhabdosphaera*	Total pg CaCO_3_/lorica
Tonga	88	0	0	280	0	184	0	20	360	932
	172	0	0	0	0	504	0	90	120	1058
	0	0	0	0	0	121	12	190	390	704
	2000	868	0	0	0	0	0	0	0	2869
	0	0	0	0	0	640	0	50	1500	2190
	96	0	0	200	0	416	144	60	300	1216
	0	0	16	0	0	680	0	10	660	1366
	248	0	0	0	0	256	0	90	0	594
	96	0	0	0	0	264	0	135	390	885
Total (*n* = 9)	366±672	1 of 9	1 of 9	2 of 9	0	330 ± 233	2 of 9	80±56	413±432	1312±706
Coral Sea (*n* = 1)	88	0	0	400	880	88	22	0	0	1472

### Dictyocysta lepida

**Figs 25–30:**
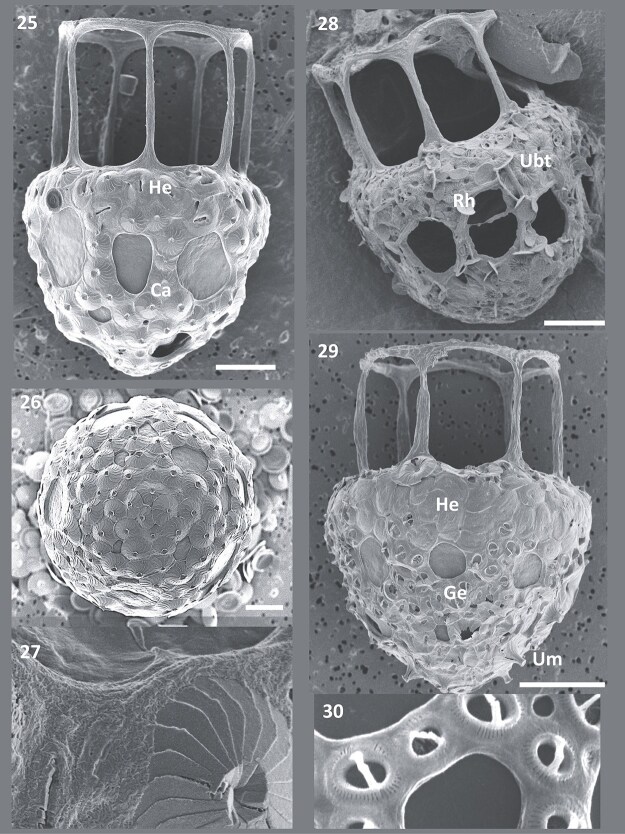
Figs 25–27: Scanning electron micrographs of *Dictyocysta lepida* from the SOTS station. Fig. 25. Bowl heavily agglutinated with *Calcidiscus* (Ca) with *Helicosphaera* (He) only at the top of the bowl. Fig. 26. Bottom view of lorica showing symmetrical coverage of liths. Fig. 27. High magnification of *Calcidiscus* lith deeply embedded on to the lorica. Fig. 28: Lorica of *D. lepida* from Tonga waters agglomerated with mostly *U. tenuis* (Ubt) and *Rhabdosphaera* (Rh) liths. Fig. 29: Lorica from the Indian Ocean with *Helicosphaera, Gephyrocapsa* (Ge) and *Umbellosphaera* in both proximal (top of bowl) and distal positions (bottom of bowl). Fig. 30: Detail of lorica of the related *Dictyocysta spinosa* from the Coral Sea with deeply embedded *Gephyrocapsa oceanica* liths onto the bowl. Scale bars: 10 μm (Fig. 26); 15 μm (Figs 25, 28, 29).

*Dictyocysta* species represent among the most elaborate tintinnid lorica architectures known, composed of a goblet-shaped bowl, with a tall cylindrical collar, 35–41 μm oral diameter, consisting of beams surrounding 6–10 large windows in one or several rows (Figs 25–29). We here focus on the ubiquitous *Dictyocysta lepida* Ehrenberg (*Dictyocysta elegans* Ehrenberg subsp. *lepida* sensu Balech, 1959) with the collar made up of a single row of windows (Figs 25, 28, 29). The rounded bowl carried 6–7 large subcircular fenestrae covered by a membrane, but sometimes (3 out of 18 loricae) completely obscured by agglomerating coccoliths. With other individuals, coccoliths could be seen deeply embedded in the framework comprising the wall of the bowl (Fig. 27). *D. lepida* loricae from the SOTS station in May 2023 (Fig. 1, location 3) carried predominantly (80–100% of area) *Calcidiscus* liths, with sparse *Helicosphaera* also present (Fig. 25). Total coccolith weight for this cold water population was 19 922 ± 5 287 pg (*n* = 18). Loricae from tropical Tonga waters (Fig. 1, location 4) were more diversely ornamented with mostly *Umbellosphaera* and *Rhabdosphaera* (Fig. 28), or *E. huxleyi* (75%) with *Umbellosphaera* and sparse *Calciosolenia* (not shown). Loricae from the Indian Ocean (Fig. 1, location 6; Fig. 29) carried *Umbellosphaera, Helicosphaera* and *Gephyrocapsa oceanica.* The related species *Dictyocysta spinosa* Kofoid and Campbell from the Coral Sea (Fig. 1, location 5) exhibited exclusively *G. oceanica* coccoliths deeply embedded on the bottom of the bowl (Fig. 30). Tropical loricae carried significantly less coccolith weight 2428 ± 2141 pg per cell (*n* = 8), approximately eight times less than their cold-water counterparts (Table V).

**Table V:** *Biogenic particles (% of total liths) agglomerated on the loricae of* Dictyocysta lepida. *Estimated as pg CaCO_3_/lorica for individual coccoliths and as total liths*.

	*Emiliania huxleyi*	*Gephyrocapsa*	*Calcidiscus leptoporus*	*Umbilicosphaera foliosa*	*Umbellosphaera tenuis*	*Helicosphaera carteri*	*Calciosolenia*	*Rhabdosphaera*	Total pg CaCO_3_/lorica
Southern Ocean Time Station (*n* = 18)	1 of 18	0	155 ± 46	0	0	10 of 18	0	0	19 922 ± 5 287
Indian Ocean (*n* = 5)	46 ± 63	2 of 5	0	2 of 5	23 ± 15	17 ± 17	0	0	3 248 ± 2 299
Tonga (*n* = 3)	74 ± 69	0	0	0	35 ± 17	0	1 of 3	1 of 3	2 428 ± 2 141

**Fig. 31:**
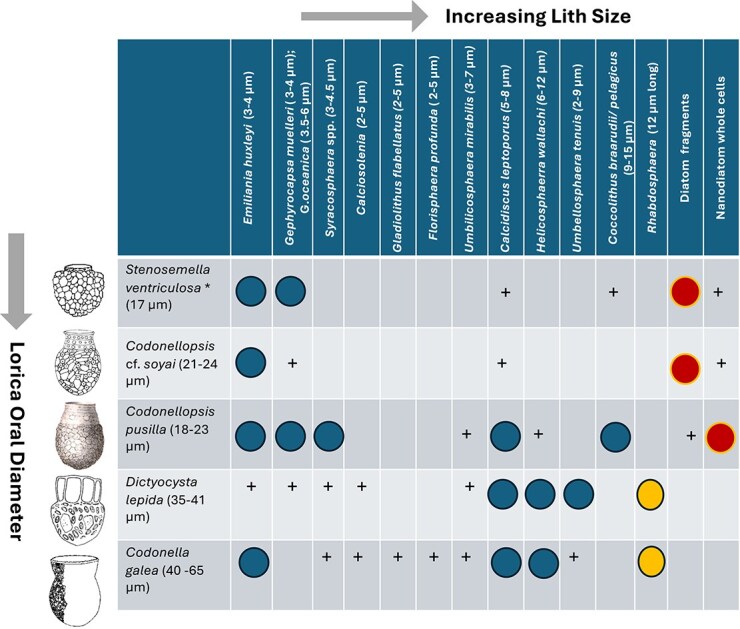
Summary diagram of tintinnid agglomerations with coccoliths and diatom or diatom fragments examined in the present study, supplemented with observations on *Stenosemella* by Rassoulzadegan (1980), Winter *et al.* (1986), Gowing and Garrison (1992) and Henjes and Assmy (2008). The five tintinnid taxa studied are arranged according to increasing lorica diameter (left) for the specimens observed, and agglomerating coccoliths are similarly arranged according to increasing lith size (top). Large blue circles mark tintinnid preferences for coccolith species, red circles designate the ability to use diatom whole cells or diatom fragments, while the yellow circles indicate the use of large prolate rhabdoliths exclusively by the two largest tintinnids; + indicate present.

Figure 31 summarizes the identity and abundance of agglomerating coccoliths and diatoms for the five tintinnid species investigated in this study, in which we included comparable quantitative observations previously published for *Stenosemella* by Winter *et al.* (1986) and Henjes and Assmy (2008). Tintinnid taxa studied are arranged according to increasing lorica diameter (as an important correlate for prey size and feeding rates; Dolan, 2010), and coccoliths are similarly arranged according to increasing lith size.

## DISCUSSION

### Taxonomy of *C. pusilla, C. soyai* and *C. galea*

The small tintinnid *C. pusilla* (Cleve) Kofoid and Campbell, 1929 has been widely reported from diverse localities such as the Subarctic (Cleve, 1899), Antarctic (Sassi and Melo, 1986; Petz, 2005) and even the Mediterranean (Balech, 1959, his Figs 81–84). This points to either an extremely eurythermal species but more likely taxonomic confusion. *Codonellopsis pusilla* was originally described from Newfoundland Banks (at water temperatures of 9–14°C) and placed in *Codonella* (Cleve, 1899) but later formally transferred to *Codonellopsis* by Kofoid and Campbell (1929, p.87) because of its a high annular collar distinctively set off from the bowl. This transfer is often credited to Jörgensen (1924) who described the new genus *Codonellopsis* (but without designating a type species), and while he mentioned in the text *C. pusilla* var.*ovata* (p.98) and *Codonella pusilla* (p.101), this taxon is not discussed, no basionym is provided, and no formal transfer instigated. This species was described as having an obovoid “clavate” bowl, 14–15 μm oral diameter, 35–48 μm long, with a cylindrical, annulated collar with “a few transverse rings”. Fenestrae were not illustrated (present type illustration Fig. 11) nor mentioned by Cleve (1899) but could have been easily overlooked. Critically, the surface of the lorica was described as “with coarse rounded pores, some of which are hexagonally framed”. This distinctive feature fits our material from the North Atlantic (Fig. 14). Balech (1959) in his treatise of Mediterranean tintinnids referred to *C. pusilla* as a highly variable species and pointed to similarities with *C. contracta* Kofoid and Campbell, which was described as having a wall with “secondary structure (Kofoid and Campbell, 1929, Fig. 147) and with “one or more elliptical fenestrae in the lower part of the spiral” (present Fig. 12). The key difference between these two taxa is the more inflated bowl and hemispherical aboral end of *C. contracta.* It has not been widely recognized however that dense biogenic agglomerations can significantly obscure and change the shape and width of the bowl (compare Figs 14 and 16). Balech (1959) also pointed out that the warm-water Mediterranean forms he identified as *C. pusilla* could well belong to the poorly defined taxon *Codonellopsis monacensis* (Rampi) Balech, originally described from Mediterranean waters as *Stenosemella monacense* (Rampi, 1950, p.4, Fig. 1).

By contrast, our material from the Kerguelen Island Plateau did not match *C. pusilla* but most closely fits *C. soyai* Hada described from the Indian sector of the Southern Ocean (Hada, 1970). Diagnostic features were the flask-shaped lorica with ovoid bowl and subcylindrical collar. Critically the surface of the obovoid bowl was described as smooth (not with a hexagonal pattern as in *C. pusilla*), The collar was flared at the oral end, composed of 3–5 spiral turns “with many small ovate fenestellae”. As in *C. pusilla.* The surface of the bowl was described as having “some coccoliths as adhering foreign particles” but Hada (1970, Fig. 48) illustrated in great detail a striking pattern that retrospectively could be interpreted as agglomerated diatom or diatom fragments (compare present Figs 2 and 3). In our Antarctic material we observed both longer (49–52 μm, Fig. 3) and shorter loricae (33–44 μm, Fig. 4), but with identical oral diameter, transdiameter and agglutination patterns. We did not attempt to separate these morphotypes. The lorica lengths of our longer morphotype matched that reported by Hada, 1970 (50–52 μm). Petz (2005) reported both *C. soyai* and *C. pusilla* from the Antarctic, but also suggested *C. soyai* to be probably conspecific with *C. pusilla* with which we do not agree. In addition to striking differences in number of fenestrae in the collar rings, the distinctive agglomerations of coccoliths and diatom fragments documented here between these two taxa also support their discrimination. The variability of lorica dimensions and shapes of smaller tintinnids remain poorly defined (summarized in Supplementary Table 1). At present no molecular sequences are available for these or related taxa (Bachy, 2012), which is critical to resolve the biogeography of these small tintinnids.

The tropical tintinnid we here refer to as *C. galea* agrees in all respect with the emended description by Kofoid and Campbell (1939) of *C. galea* of 78–120 μm lorica length (emended from Kofoid and Campbell, 1929), except for the incidence of smaller cells of 60–113 μm length (77 average ± 16 μm standard deviation). We note that other authors similarly included smaller forms under this name such as 54–120 μm long (Marshall, 1969) and 73–100 μm long (Komarovsky, 1959, from the Red Sea). Critically, Kofoid and Campbell, 1939 described by LM comparable coccolith agglutination as we observed from *Umbilicosphaera, Syracosphaera mediterranea, Rhabdosphaera clavigera* (except for not being able to resolve the smaller *Florisphaera* and *Gladiolithus*-like liths, which we detected by SEM).

### Do tintinnids have a preference for particular coccoliths?

In the present work, we investigated tintinnid populations for which we had suitable material available in terms of numbers of individuals, preservation and unambiguous tintinnid species identifications to semi-quantitatively assess lorica variations at a single location. We here address tintinnids that primarily utilize coccoliths rather than diatoms, which we will expand on in a separate communication. Wasik *et al.* (1996) previously documented abundance and species compositions of diatoms adhering to the loricae of Antarctic *Laackmaniella naviculaefera, Codonellopsis gaussi, C.balechi* and Baltic Sea *Tintinnopsis lobiancoi.* These authors concluded that certain diatoms (*Fragilariopsis, Thalassiosira*) were actively selected and it is not a rule that tintinnids only use those which are dominant. Agglomerations of morphologically variable diatoms and diatom fragments are more challenging to quantify than coccolith agglomerations, also calling for attention to the attachment of living diatoms in a possible symbiotic association (e.g. Armbrecht *et al.,* 2017; Gómez, 2020). The summary diagram in Fig. 31 demonstrates how the two smallest, cold-water tintinnids *Stenosemella* and *Codonellopsis* cf. *soyai* (17–23 μm oral diameter) appeared to non-selectively use the ubiquitous smallest coccolithophorids *Emiliania huxleyi* or *Gephyrocapsa muelleri* (3–4 μm lith length), most often attached to the lorica with the proximal shield. They rarely attached sparse larger liths such as *Calcidiscus* or *Coccolithus* (the latter illustrated by Winter *et al.,* 1986 for *Stenosemella* from the Agulhas Current). However, when coccolithophorids were sparse or absent, these tintinnids shifted to using almost exclusively ubiquitous diatom fragments. This shift was first demonstrated by Henjes and Assmy (2008) for *Stenosemella* from the Antarctic Polar Front, and here was also suggested for *C.* cf. *soyai* from the Kerguelen Plateau. Admittedly, our material did not cover time series observations nor data on ambient plankton communities. Tintinnids are thought not to be able to mechanically crush diatoms, hence in all likelihood these microzooplankton capitalize on the leftovers from active feeding on diatom blooms by larger copepods or krill. The ubiquitous nanoplankton *Emiliania* is unique in being able to produce multiple layers of coccoliths and prolifically shed liths into ambient seawater, a feature not known however from any other coccolithophorid species. This raises the question of how tintinnids are able to dismantle large coccolithophorid cells because agglomerated liths are almost always intact. The intermediate sized tintinnid *C. pusilla* from the North Atlantic and the Southern Ocean*,* oral diameter 20–23 μm but up to 35 μm bowl length, was able to use small *Emiliania, Gephyrocapsa* and *Syracosphaera* liths but if available appeared to preferentially select larger and heavier *Calcidiscus* and *Coccolithus* liths (5–15 μm diameter). Ambient water column coccolithophorid assemblages in the North Atlantic (APERO cruise) were comprised in decreasing order of numerical abundance of *Emiliania*, *Calcidiscus*, *Gephyrocapsa muelleri*, *Syracosphaera pulchra* and *Coccolithus*. Similarly, coccolithophorid assemblages in sediment traps at the SOTS station were dominated by *Emiliania,* with *Calcidiscus* at most 20% of total coccolithophores and *Coccolithus* rare (Findlay and Giraudeau, 2000; Rigual Hernández *et al.,* 2020). Diatom fragments were rarely used by this tintinnid species but more common was the agglutination on the top of the bowl of small intact diatom cells of *Fragilariopsis pseudonana* (4–5 μm) or *Nitzschia bicapitata* (14 μm long). The latter has also been reported from, e.g. *Acanthostomella minutissima* from New Zealand (Burns, 1983). This strategy constitutes a transition to tintinnids such as *C. gaussi, Laackmanniella* and *Tintinnopsis* that exclusively use diatoms (Wasik *et al.,* 1996) many of them alive (Armbrecht *et al.,* 2017). Of interest is that the freshwater *Codonella cratera* Leidy, thriving in lake environments without coccolithophorids, uses small discoid diatoms such as *Cyclotella ocellata* (5 μm diameter) of a size and shape comparable to *Emiliania* liths (Bettighofer, 2024). Similarly, small *C. pusilla* like loricae from the Mediterranean have been observed to carry mixtures of *Emiliania/Gephyrocapsa* coccoliths as well as minute (3 μm diameter) *Minidiscus* diatom cells (image available at https://ecotaxa.obs-vlfr.fr/prj/15396). This strongly points to size selection of biogenic materials irrespective of their taxonomic identity.

Not surprisingly, the largest tintinnids studied here, *D. lepida* and *C. galea,* 35–65 μm oral diameter, housing the strongest swimming ciliates, exhibited the capacity to capture the greatest biodiversity of liths, again with a strong preference for the larger coccoliths of *Calcidiscus, Helicosphaera, Coccolithus, Umbellosphaera* and also the ability to capture *Rhabdosphaera* liths (up to 12 μm long). This unique latter feature was already noted by Kofoid and Campbell (1939) for *C. galea* and *Codonaria australis* (Kofoid and Campbell) Kofoid and Campbell, and confirmed by Takahashi and Ling (1984) also for *Codonellopsis minor* (Brandt) Kofoid and Campbell and by Bachy (2011) for *Ceratocephala orthoceras* (Haeckel) Jörgensen. Such prolate liths are preferentially positioned at the bottom of the bowl (Figs 24 and 28). Similarly, with *D. lepida* the same funnel-shaped *Umbellosphaera* liths could be positioned in distal view on top of the bowl, but deposited in proximal view at the bottom of the bowl (Fig. 29) with the protruding stems adding to surface roughness. Small lighter liths (e.g. *Florisphaera, Gladiolithus*) and diatoms tended to be positioned mostly close to the mouth of the bowl (Figs 17 and 24). On some loricae, e.g. of *Codonellopsis elongata* from Palau (Young *et al.,* 2022, JRYSEM-275-22) the coverage of spiraling rows of asymmetrical *Helicosphaera* liths strongly resembles the spiraling dynamics of ciliar beating. On other loricae heavier liths can be deposited on the middle and leaving the bottom of the bowl for smaller liths (Figs 16 and 19). These patterns suggests hydrodynamic sorting of liths jettisoned away from the cytostomal region by ciliar beating.

### Possible function of agglomerated biogenic particles

Numerous explanations have been proposed for the functions of agglomerated tintinnid loricae, including: (i) protective armor to reduce its susceptibility to copepod predation; (ii) ballast to quickly sink away from predators; (iii) enhancing capture of food particles around the oral cilia, and (iv) modification of swimming behavior by tethering to detritus (Jonsson *et al.,* 2004) or diatom frustules (reviewed by Agatha *et al.,* 2013). Limited data on sinking rates of tintinnids (Suzuki and Taniguchi, 1995) indicate that small hyaline loricae such as *Acanthostomella* sink at 0.25–2.08 m day^−1^ while two species with agglutinated loricae, *Tintinnopsis ampla* Hada and *Tintinnopsis beroidea* Stein exhibited sinking rates of 1.90 and 15.9 m day^−1^. The latter was covered with coccolithophorids, mostly *Emiliania*. Takahashi (1982) provided an estimate for the dry weight of loricae from a mixture of the medium-size *Codonellopsis minor* and larger *C. biedermanii* (Brandt) Kofoid and Campbell as 40 000 ± 20 000 pg per individual. From published images by Henjes and Assmy (2008) we estimated a coccolith load for the smallest tintinnids *Stenosemella* of 1500 to 1700 pg and for our *C. cf. soyai* of 880–1488 pg. By contrast, for *C. pusilla* in the Southern Ocean ballasted by heavy *Calcidiscus,* this could add an extra 10 540 ± 3537 pg (i.e. an extra 25% of lorica weight). By contrast, ballasting of the large tropical Tonga *C. galea* was insignificant at 1312 ± 3706 pg. Similarly, tropical Indian Ocean tropical *D. elegans* loricae carried significantly less coccolith weight of 2428 ± 2141 pg per cell, compared to their Southern Ocean counterparts of 19 922 ± 5287 pg. These observations suggest that tintinnid lorica agglomerations may be quantitatively more important in more viscous colder waters compared to less viscous warmer tropical waters. Agglomerations with solid calcium carbonate coccoliths consistently added more weight to tintinnid loricae than empty broken siliceous diatom frustules could achieve (Tables II–V). Lorica agglomerations however may serve purposes other than ballasting, which has also been suggested as a strategy to sink away from predators. At low Reynolds numbers seawater is viscous and the effect of lorica surface roughness on swimming speed has not yet been explored. Instead, most studies on ciliate swimming behavior have all been confined to the smooth-loricate tintinnids *Favella* and *Tintinnopsis* or aloricate ciliates *Strombidium* and *Strombilidinium* (Montagnes, 2013).

Feeding and swimming in tintinnids are tightly linked by their cilia creating feeding currents that bring prey to the cytostomal region. Tintinnids can modify feeding behavior by modulating cilia kinematics (Wandel and Holzman, 2022). Probability of prey encounter increases with predator size, prey size and density. Tintinnids prefer prey of roughly 20–30% of the size of their lorica diameter (Dolan *et al.,* 2013) and prefer spherical instead of prolate particles. The preferential capture in our study by larger tintinnids of larger discoid *Calcidiscus, Coccolithus* and *Helicosphaera* liths and rarely prolate *Rhabdosphaera* liths confirm this. By contrast the smaller tintinnids appeared to use either abundant ambient small *Emiliania* liths or diatom fragments more passively (Fig. 31). When captured, prey may be rejected, or moved into the cytostomal region, where they are processed and digested, and indigestible parts egested. Whether agglomerated particles are rejected food particles or directly captured onto sticky loricae is not clear. Typical intact coccospheres of *Emiliania* are 5–12 μm in diameter (Henderiks, 2008), which is too big for ingestion by *Stenosemella, C. soyai* or *C. pusilla.* However, coccosphere diameters for *Calcidiscus* are 10–13 μm, for *Coccolithus* 10–30 μm and *Helicosphaera* 9–16 μm, which is in the range of capture for the larger *D. lepida* and *C. galea*. On the other hand, the coccosphere diameter of *Rhabdosphaera* of 20–35 μm is too much even for these larger tintinnids. Tintinnids that build agglomerated loricae offer fascinating opportunities for analysis by high-speed videography of feeding and swimming (Wandel and Holzman, 2022) and by using photogrammetry of surface roughness to estimate drag coefficients (Barton *et al.,* 2010). The next step should be to challenge cultured or hand-picked tintinnids from freshly collected field samples with mixtures of microalgal cultures and fluorescent beads of different sizes, shapes and weights to advance our understanding of the ecological advantages of tintinnid lorica agglomeration.

## Supplementary Material

Hallegraeff_Agglomerated_tintinnids_Supplementary_Table_1_fbaf030

## Data Availability

All SEM images used in this work have been deposited on a dedicated Ecotaxa platform focused on tintinnid loricae with agglomerated biogenic particles (https://ecotaxa.obs-vlfr.fr/prj/15396). We encourage other workers to add their images of agglomerated tintinnid loricae to stimulate collaboration.
