# Supplementary material for: Agglomerated loricae of the tintinnids Codonella, Codonellopsis and Dictyocysta from North Atlantic, tropical Pacific and Southern Ocean waters
Source: J Plankton Res. 2025 Aug 12;47(5):fbaf030. doi: 10.1093/plankt/fbaf030 (PMC12343039; doi:10.1093/plankt/fbaf030)
Supplement: Hallegraeff_Agglomerated_tintinnids_Supplementary_Table_1_fbaf030 [file hallegraeff_agglomerated_tintinnids_supplementary_table_1_fbaf030.docx]

Supplementary Table 1. Summary of diagnostic features of the tintinnid *Codonellopsis pusilla* and similar species

| Species | Type locality  (temperature) | Type illustration | Dimensions | Diagnostic features |
| --- | --- | --- | --- | --- |
| *Codonellopsis pusilla* (Cleve) Kofoid & Campbell 1929 | Subarctic  (Newfoundland Banks); 9-14^o^C | Cleve 1900,  p.970, fig.[3] | 14-15 µm oral diameter;  35-48 µm long; 33 µm width | Obovoid clavate bowl, with cylindrical annulated collar with a few transverse rings. Surface of the lorica with coarse rounded pores, some of which hexagonally framed. |
| **Present Material**  North Atlantic |  |  | 18-23 µm oral diameter; 29-35 µm wide; 32- 35 µm bowl length | Sparse fenestrae newly recognised (SEM, Fig.14-17). Surface of lorica matches type description (SEM, Fig. 14). Transparent collar of 4-5 rings. |
| Mediterranean; Balech 1959, Figs 81-84 |  |  | 16-17 µm oral diameter, 38-45 µm length, 29-32.5 µm width | Highly variable species, mostly related to lateral flattening. Hexagonal surface structure of bowl. Sparse fenestrae, sometimes absent.  Does not agree exactly with *C. pusilla* and bears great resemblance to C. *contracta.* |
| *Codonellopsis (Stenosemella) monacense* (Rampi) Balech 1959 | Mediterranean | Rampi 1950, p.4, Fig.1 | 18-20 µm oral diameter; 37-40 µm length, 30-33 µm wide | Oviform lorica with slightly pointed aboral area; low neck with slightly curved sides on the outside. Surface of the lorica covered by an almost regular alveolar structure, smooth or slightly scrobulated. |
| *Codonellopsis contracta* Kofoid & Campbell 1929 | Peruvian Current | Kofoid & Campbell 1929, Fig. 147 | 42-48 µm length | Lorica wall with secondary structure, with one or more elliptical fenestrae in the lower part of the spiral. Differs from *C.pusilla* in the more inflated bowl and hemispherical aboral end. |
| *Codonellopsis soyai* Hada 1970 | Indian sector  of Southern Ocean; -1.7 to-1.4^o^C | Hada 1970,  Fig. 48. | 20-21 µm oral diameter, 50-52 µm length, 32-38 µm width | Flask-shaped lorica with ovoid bowl and subcylindrical collar. Surface of the bowl is smooth. Hyaline collar as long as 1/3 of total length, flared at the oral end, composed of 3-5 spiral turns with many small ovate fenestellae. |
| **Present Material**  Kerguelen Island Plateau |  |  | 21-24 µm oral diameter; 32-40 µm wide; longer (49-52 µm, and shorter loricae (33-44 µm) co-occurred _ | Newly recognised diatoms as agglomerating particles (SEMs, Figs.6-10 |
